# Cannabidivarin alleviates neuroinflammation by targeting TLR4 co-receptor MD2 and improves morphine-mediated analgesia

**DOI:** 10.3389/fimmu.2022.929222

**Published:** 2022-08-10

**Authors:** Xue Wang, Cong Lin, Siru Wu, Tianshu Zhang, Yibo Wang, Yanfang Jiang, Xiaohui Wang

**Affiliations:** ^1^ Department of Anesthesiology, The First Hospital of Jilin University, Changchun, China; ^2^ Laboratory of Chemical Biology, Changchun Institute of Applied Chemistry, Chinese Academy of Sciences, Changchun, China; ^3^ School of Applied Chemistry and Engineering, University of Science and Technology of China, Hefei, China; ^4^ Key Laboratory of Organ Regeneration and Transplantation of the Ministry of Education, Genetic Diagnosis Centre, The First Hospital of Jilin University, Changchun, China; ^5^ Beijing National Laboratory for Molecular Sciences, Beijing, China

**Keywords:** Toll-like receptor 4, myeloid differentiation protein 2, cannabidivarin, neuroinflammation, analgesia, pain

## Abstract

Toll-like receptor 4 (TLR4) is a pattern-recognition receptor (PRR) that regulates the activation of immune cells, which is a target for treating inflammation. In this study, Cannabidivarin (CBDV), an active component of Cannabis, was identified as an antagonist of TLR4. *In vitro*, intrinsic protein fluorescence titrations revealed that CBDV directly bound to TLR4 co-receptor myeloid differentiation protein 2 (MD2). Cellular thermal shift assay (CETSA) showed that CBDV binding decreased MD2 stability, which is consistent with *in silico* simulations that CBDV binding increased the flexibility of the internal loop of MD2. Moreover, CBDV was found to restrain LPS-induced activation of TLR4 signaling axes of NF-κB and MAPKs, therefore blocking LPS-induced pro-inflammatory factors NO, IL-1β, IL-6 and TNF-α. Hot plate test showed that CBDV potentiated morphine-induced antinociception. Furthermore, CBDV attenuated morphine analgesic tolerance as measured by the formalin test by specifically inhibiting chronic morphine-induced glial activation and pro-inflammatory factors expression in the nucleus accumbent. This study confirms that MD2 is a direct binding target of CBDV for the anti-neuroinflammatory effect and implies that CBDV has great translational potential in pain management.

## Introduction

The innate immune is the first line of defense against bacterial infections of the immune system (1). Innate immune responses, such as inflammation, are mediated by pattern-recognition receptors (PRRs) that recognize pathogen-associated molecular patterns (PAMPs). Toll-like receptors (TLRs) are the first identified PRRs to trigger innate immune responses (2, 3). TLR4 is a key member of the TLRs family, which forms a complex on the cell surface with myeloid differentiation protein 2 (MD2) (4). Lipopolysaccharide (LPS), a significant component of the outer wall of Gram-negative bacteria, is the natural ligand of TLR4 as PAMPs (5). Besides for PAMPs, TLR4 also recognizes damages-associated molecular patterns (DAMPs) and xenobiotic-associated molecular patterns (XAMPs) (5). The high mobility group box 1 (HMGB1), heat shock protein 70 (HSP70) and the myeloid-related protein 8 (MRP8) are the endogenous ligands of TLR4 as DAMPs (6). Some psychoactive compounds, such as morphine, act as XAMPs to activate the TLR4 signaling pathway and create neuroinflammation (7–11). Morphine induces glia activation (12) and creates a neuroinflammatory response within the central nervous system (CNS), compromising morphine-induced analgesia as well as contributing to morphine-induced analgesic tolerance (7, 13, 14). Therefore, TLR4 antagonists could be potential agents for enhancing morphine analgesic efficacy and preventing morphine tolerance (9, 15). Numerous TLR4 small-molecule inhibitors have been developed (16). However, no TLR4 antagonists have been approved for clinical use.

Cannabidivarin (CBDV) is a safe, non-psychoactive phytocannabinoid isolated from *Cannabis Sativa* (17). Although CBDV is usually a minor constituent of naturally-occurring cannabinoids found in cannabis, CBDV has been attracting great interest owing to its potential benefits to clinical conditions that cannabidiol (CBD) cannot effectively treat (18, 19). Owing to its lipophilicity and blood-brain barrier (BBB) penetrability (20, 21), CBDV has recently gained much attention as for its ability to modulate neurological diseases (17, 22, 23). Considering TLR4 is a XAMPs receptor for surveiling foreign substances in CNS, it would be interesting to explore whether CBDV acts as a XAMP and can be sensed by MD2, an accessory protein of TLR4 responsible for the recognition of ligand. Herein, MD2 was found as a direct target of CDBV. CBDV inhibited TLR4 signaling NF-κB and MAPK signaling axes and the downstream pro-inflammatory factors. Moreover, CBDV improved morphine-induced analgesia by specifically inhibiting chronic morphine-induced glial activation and pro-inflammatory factors expression in the nucleus accumbent (NAc). This study implies that CBDV has great translational potential in pain management.

## Manuscript formatting

### Materials and methods

#### Materials

Microglial BV-2 cells were obtained from the China Center for Type Culture Collection. CBDV, crystal violet, 2, 3-diaminonaphthalene and paraformaldehyde were purchased from Sigma-Aldrich. Morphine was obtained from China National Institutes for Food and Drug Control. Ultrapure LPS, HEK Blue hTLR4 cells, and HEK-Blue Selection were purchased from *In vivo*gen. The Phospha-Light™ SEAP Reporter Gene Assay System was purchased from Applied Biosystems. The Dual-Glo Luciferase Assay System was purchased from Promega. Hifair III 1st Strand cDNA Synthesis SuperMix for qPCR and HieffqPCR SYBR Master Mix were obtained from Yeasen Biotech Co., Ltd. PCR primers were purchased from Comate Bioscience Co., Ltd. Cell lysis buffer for western blotting was purchased from Beyotime Biotechnology. Primary antibodies targeting GFAP, p38 MAPK, NF-κB p65, ERK (1/2), IKKβ, SAPK/JNK, phospho-NF-κB p65, phospho-ERK (1/2), phospho-SAPK/JNK, phospho-IKKα/β, phospho-p38 MAPK, goat anti-rabbit HRP-linked antibody and goat anti-mouse HRP-linked antibody were purchased from Cell Signaling Technology. The anti-Iba-1 antibody was purchased from Affinity Bioscience. 4’, 6-diamidino-2-phenylindole (DAPI) was purchased from Absin Bioscience Inc.

#### Fluorescence titrations of MD2 with CBDV

The method of fluorescence titrations of MD2 with CBDV was performed as described (24).

#### Cellular thermal shift assay (CETSA)

CETSA assay was performed as described (24).

#### *In silico* simulation

The crystal structure of MD2 was extracted from the mouse TLR4/MD2 complex (PDB ID: 2Z64) (25). Autodock Vina 1.1.2 was used for molecular docking in a box of 46 × 58 × 53 Å^3^ with default settings (26). MD2 was considered rigid and CBDV was treated as semi-flexible during the docking process. Twenty docking poses were generated and the best docking pose was selected for further simulations.

Apo-MD2 and MD2-CBDV were further investigated through molecular dynamics (MD) simulations performed by the Gromacs 2021.2 program (27, 28). The CHARMM36m force field was used for proteins (29, 30). The ligand parameters were generated by the Antechamber tool (31). All solutes were solvated in a TIP3P water box with 0.15 M NaCl to mimic the physiological condition. The systems were equilibrated in the isothermal-isobaric (NPT) ensemble at a temperature of 310 K for 100 ns. The SHAKE algorithm was applied to restrain all bonds involving hydrogen (32). The particle-mesh Ewald (PME) summation method was applied to treat long-range electrostatic interactions (33). The pressure was set at 1 atm with the Nosé-Hoover Langevin piston method (34).

The RMSD (root-mean-square deviation) and RMSF (root-mean-square fluctuation) analyses were performed through MD Analysis (35). The binding energy (enthalpy) and per-residue energy contributions were calculated by the molecular mechanics/Poisson-Boltzmann (generalized-Born) surface area method with the gmx_MMPBSA tool (36, 37). Each system was repeated three times independently. The interactions between MD2 and CBDV were shown by PyMol (38).

#### Immunoblotting

Immunoblotting was performed as described (24). BV-2 cells were seeded at 5 × 10^5^ cells/well in 6-well plates. After 24 h incubation, cells were treated with 200 ng/mL LPS and indicated concentrations of CBDV for 1 h. The cells were lysed in RIPA buffer with complete protease inhibitor cocktail and phosphatase inhibitors. Equal amounts of protein (20-40 μg) were separated by SDS-PAGE and transferred to PVDF membranes. After blocking with 5% non-fat dry milk for 1 h, membranes were incubated with corresponding primary antibodies (All primary antibodies were diluted at 1: 1000) overnight at 4°C. After washing three times in Tris-buffered saline with 0.1% Tween 20 (TBST) for 5 min each time, membranes were incubated with secondary antibody-HRP conjugate (1: 3000) for 1 h at room temperature. After washing three times in TBST, the membranes were detected using Tanon-5200 Multi. Image J was used for later densitometric analysis.

#### Nitric oxide (NO) assay

BV-2 cells were seeded in 96-well plates at a density of 4 ×10^4^ cells per well and cultured in Dulbecco’s modified Eagle’s medium (DMEM) with 10% fetal bovine serum (FBS), 50 U/mL penicillin, and 50 μg/mL streptomycin for 24 hours at 37°C in a 5% CO_2_ incubator. After 24 hours, the media was replaced with DMEM alone and treated with LPS (200 ng/mL) and the indicated concentrations of CBDV. The supernatant was transferred to another 96-well plate and determined by the 2, 3-diaminonaphthalene-based fluorescent method as described (24).

#### Secreted embryonic alkaline phosphatase (SEAP) assay

SEAP assay was performed as described (7). Briefly, HEK Blue hTLR4 cells were cultured in DMEM supplemented with 10% FBS, 50 unit/mL penicillin, 50 μg/mL streptomycin and 1 × HEK-Blue selection. Cells were seeded in 96-well plates at a density of 4 ×10^4^ cells per well. After 24 h incubation, the medium was replaced with Opti-MEM supplemented with 0.5% FBS, 50 unit/mL penicillin, 50 μg/mL streptomycin and 1 × non-essential amino acid. Cells were treated with 20 ng/mL LPS and indicated concentrations of CBDV. After 24 h incubation, NF-κB activity was measured through the Phospha-Light SEAP Reporter Gene Assay System according to the manufacturer’s instructions.

#### Dual-luciferase NF-κB reporter assay

Dual-luciferase NF-κB reporter assay was performed as described (7). Briefly, BV-2 NF-κB luciferase reporter cells were cultured in DMEM supplemented with 10% FBS, 50 unit/mL penicillin, 50 μg/mL streptomycin and 2 μg/mL puromycin. Cells were seeded in 96-well plates at a density of 1 ×10^4^ cells per well. After 24 h incubation, the medium was replaced with Opti-MEM supplemented with 0.5% FBS, 50 unit/mL penicillin, 50 μg/mL streptomycin and 1 × non-essential amino acid). Cells were treated with 200 ng/mL LPS and indicated concentrations of CBDV. After 24 h incubation, NF-κB activity was measured through Dual-Glo Luciferase Assay System according to the manufacturer’s instructions.

#### Cell viability assays

Cellular viability was determined by the crystal violet staining method and Cell Counting Kit-8 (CCK-8) assay. Crystal violet staining was performed as described in the following. BV-2 NF-κB luciferase reporter cells were cultured and treated as indicated in the dual-luciferase NF-κB reporter assay. After 24 h treatment, cells were fixed with 100 μL 4% paraformaldehyde for 5 minutes and then stained with 100 μL 0.05% crystal violet for 15 minutes. After washing three times with water, 200 μL ethanol was added for each well and incubated for 20 min at room temperature. Absorbance at 540 nm was measured using a SYNERGY H1 Microplate Reader.

For CCK-8 assay, HEK Blue hTLR4 cells were cultured and treated as indicated in the SEAP assay. HEK Blue hTLR4 cells were treated in 96-well plates. After 24 h treatment, 20 μL CCK-8 assay solution was added for each well and cells were incubated in a 5% CO2, 37°C incubator for 2 h. Absorbance was detected at 450 nm with 650 nm as the reference wavelength.

#### qRT-PCR

BV-2 cells were counted and plated in 6-well plates at a density of 4 ×10^4^ cells per well. After overnight incubation, LPS (200 ng/mL) and indicated concentrations of CBDV were added. After 24 h incubation, TRIzol was used to isolate total RNA from BV-2 cells. RNA was reverse-transcribed into cDNA. qPCR was performed subsequently using the SYBR Green method. Rpl27 was set as a reference gene. The primers sequence for TNF-α, IL-1β, and Rpl27 were shown in **Table 1**. The data were analyzed by the ^ΔΔ^Ct method.

**Table 1:** Primer sequences of IL-1β, IL-6, TNF-α and Rpl27.

Gene		Sequence (5’ - 3’)
IL-1β	Forward	CCACCTTTTGACAGTGATGA
	Reverse	GAGATTTGAAGCTGGATGCT
IL-6	Forward	TAGTCCTTCCTACCCCAATTTCC
	Reverse	TTGGTCCTTAGCCACTCCTTC
TNF-α	Forward	CCCTCCAGAAAAGACACCATG
	Reverse	GCCACAAGCAGGAATGAGAAG
Rpl27	Forward	AAGCCGTCATCGTGAAGAACA
	Reverse	CTTGATCTTGGATCGCTTGGC

For qRT-PCR of brain tissue, nucleus accumbent (NAc) region was firstly collected as following. Animals were deeply anesthetized by intraperitoneal injection of pentobarbital (100 mg/kg) and perfused with saline. The brain was removed and NAc region was dissected. The tissues were immediately kept in liquid nitrogen. After solubilizing the tissue in Trizol using homogenizer, total RNA from the NAc was isolated. RNA was reverse-transcribed into cDNA and qPCR was performed as described above.

#### *In vivo* studies

##### Animal

Adult male BALB/c mice (weight: 20-25 g) purchased from the Liaoning Changsheng Biotech Company were employed in this study. Mice were housed 3-5 per cage at standard conditions (22-25°C, 12h: 12h light: dark cycle, free access to standard food and water). All experiments were approved by the Institutional Animal Care and Use Committee (IACUC) of Changchun Institute of Applied Chemistry, Chinese Academy of Sciences (2022–0090).

##### Hot plate assay

The temperature of the hot plate was set at 55°C. The latency period was measured by the pain threshold when the mouse lifted or licked a hind paw, jumping or vocalizing. One day before the experiment, the baseline pain threshold was measured by averaging the values of two 1 h interval measures. The pain threshold was calculated as described (7), and the cut-off duration was 30 s. Animals were randomly divided into four groups (n = 5), including the control group, CBDV group, morphine group, and morphine + CBDV group. Before the test, mice were intraperitoneally injected with CBDV (8 mg/kg or 50 mg/kg) 30 min before morphine (5 mg/kg, *i.p.*). Following morphine administration, the pain threshold was recorded at different time points. Data were expressed as percent maximum potential effect (% MPE): %MPE = (post-drug latency - baseline latency)/(cut-off time - baseline latency) × 100.

##### Formalin test

Animals were randomly divided into each group (n = 5-6). The mice had 30 min to get used to the plastic chamber with black sides and transparent bottom (12 cm ×12 cm ×15 cm). 20 μL of 2% formalin was injected into the hind paw with a 27-gauge needle described previously for the formalin test (39). CBDV (50 mg/kg) was intraperitoneally injected 1 h before the administration of formalin. Morphine (1 mg/kg, *i.p.*) was given 30 min before the injection of formalin. Once formalin had been injected, the mice returned to the chamber, and the video was recorded for 40 min. There were two phases in the formalin test: the first (acute phase) ranged from 0-10 min and the second phase (tonic phase) ranged from 10-40 min. The seconds of licking and biting of the injected paw was calculated and analyzed in each phase.

#### Immunofluorescence

The procedure for immunofluorescence staining follows the steps described previously (40). Animals were deeply anesthetized by intraperitoneal injection of pentobarbital (100 mg/kg) and perfused with phosphate-buffered solution (PBS, pH 7.4) followed by 4% paraformaldehyde. After dissection, the brain was removed and soaked in 4% paraformaldehyde overnight. Then, the brain was cryoprotected in 20% sucrose in PBS at 4°C overnight. Brain sections (40 μm thick) containing the medial prefrontal cortex (mPFC), nucleus accumbens (NAc), and ventral tegmental area (VTA) regions were obtained by a cryostat microtome. The slides were washed with 0.1 M PBS buffer three times and then incubated in primary antibodies Iba-1 (Affinity, DF6442) and GFAP (Cell Signaling Technology, 3670) at 4°C for two days. After three washes with PBS, the sections were exposed to a secondary antibody for overnight incubation. After a thorough wash in PBS, 4’, 6- diamidino-2-phenylindole (DAPI) staining was performed. Olympus VS120 microscope was used to take the fluorescent images.

#### Statistical analysis

Data were expressed as the mean ± S.E.M, and analysis of variance was carried out using one-way analysis of variance (ANOVA). All statistical analyses were performed with GraphPad Prism 8.0 and Origin 8. The statistical significance was marked above the bar for each figure. P < 0.05 was considered statistically significant in all analyses.

### Results

#### Biophysical binding of CBDV with MD2

**Figure 1:**
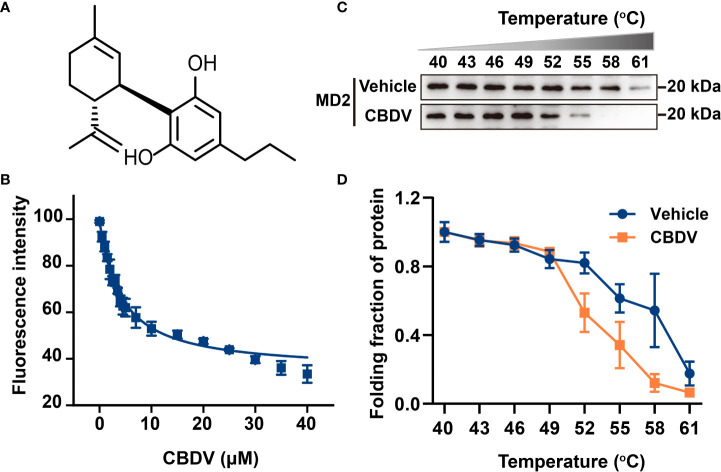
Biophysical binding of CBDV with MD2. **(A)** The chemical structure of CBDV. **(B)** Titration curve of MD2 intrinsic fluorescence with the increasing concentrations of CBDV. Emission at 337 nm (excitation at 280 nm) was plotted against the titrated CBDV concentration. K_d_ value of 3.9 ± 0.3 μM was derived by nonlinear least-squares fitting of a one-site binding model for CBDV interacting with MD2. **(C, D)** Cellular thermal shift assay (CETSA) of MD2 with CBDV. CBDV binding decreased MD2 thermal stability (ΔT_m_ = -4.3 ± 0.1 °C). All experiments were performed three times independently, and data were given as the mean ± S.E.M.

MD2 is the TLR4 co-receptor that is responsible for the recognition of ligands (41). In order to explore whether CBDV could be sensed by TLR4, the direct interaction of MD2 and CBDV (**Figure 1A**) was investigated. *In vitro* MD2 intrinsic fluorescence quenching titration with CBDV was performed (**Figure 1B**). A dissociation constant *K*_d_ of 3.9 ± 0.3 μM was derived by the nonlinear least-square fitting of the titration curve of MD2-CBDV interaction. To verify that MD2 is indeed the endogenous target of CBDV, cellular thermal shift assay (CETSA) was carried out (**Figure 1C**). The folding fraction of MD2 decreased with the increasing of temperature and CBDV binding decreased the melting temperature (T_m_) of MD2 by 4.3 ± 0.1°C (**Figure 1D**), indicating that CBDV decreased MD2 thermal stability. Taken together, these biophysical binding characterizations show that MD2 is a direct target of CDBV.

#### *In silico* simulation of CBDV interacting with MD2

**Figure 2:**
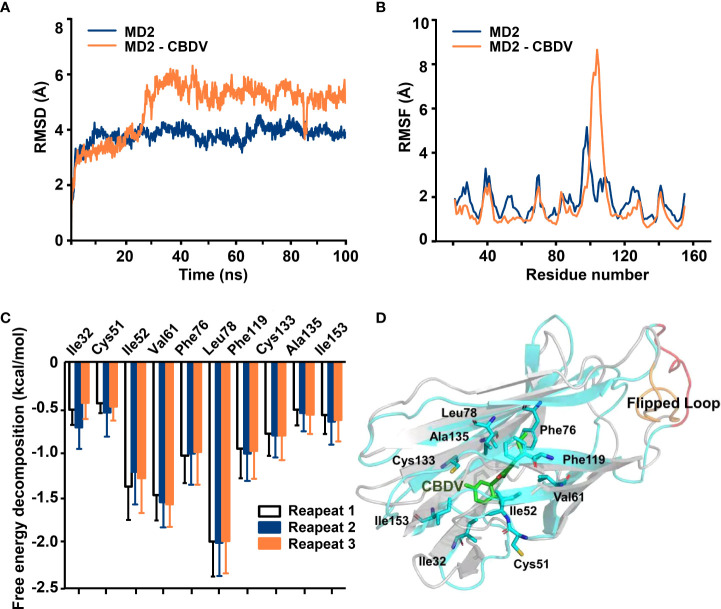
*In silico* simulation of CBDV interacting with MD2. **(A)** Time evolution of RMSDs for apo-MD2 and CBDV-bound MD2 (MD2-CBDV) during the MD simulations at 310 K. **(B)** Time evolution of RMSFs of MD2 and CBDV bound MD2 during the MD simulations at 310 K. **(C)** Per-residue energy decomposition for key residues. **(D)** The representative binding mode of CBDV with MD2 at 310 K after MD simulation. CBDV was shown as ball-stick model. MD2 in the MD2-CBDV system was shown as cartoon model (cyan) aligned to apo-MD2. Key residues of MD2 in interacting with ligands were shown as sticks. The flipped loops were labeled with orange and red in apo-MD2 and MD2-CBDV, respectively.

To investigate atomic details of the interactions between CBDV and MD2, MD simulations were performed. As shown in **Figure 2A**, the root-mean-square deviation (RMSD) values of backbone atoms of apo-MD2 and MD2 complexed with CBDV showed that both systems reached stable states in 100 ns simulations. The RMSD value of apo-MD2 stabilized at around 4.0 Å, and the RMSD value of MD2 complexed with CBDV stabilized at around 5.5 Å. Root-mean-square fluctuation (RMSF) analysis was performed (**Figure 2B**). The binding of CBDV rendered an internal loop (residues 100 - 108) of MD2 flipped out and much more flexible, indicating that CBDV destabilizes MD2. This result is consistent with the experimental CETSA data. The binding energy of CBDV to MD2 was decomposed (**Figure 2C**) and CBDV was stabilized by the interactions with Ile32, Cys51, Ile52, Val61, Phe76, Leu78, Phe119, Cys133, Ala135, and Ile153 (**Figure 2D**).

#### CBDV inhibits TLR4 signaling and LPS-induced pro-inflammatory factors

**Figure 3:**
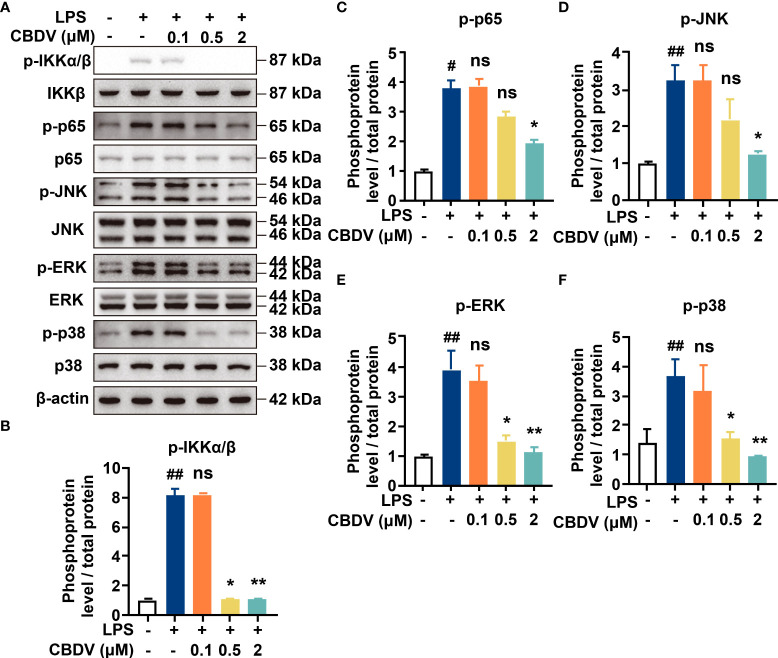
Cellular characterizations of CBDV on TLR4 signaling. **(A)** The effect of CBDV on LPS-induced TLR4 signaling as measured by western blotting. **(B-F)** The quantification of the phosphorylation of IKKβ **(B)**, p65 **(C)**, JNK **(D)**, ERK **(D)** and p38 **(F)** shown in **(A)**. All experiments were performed three times independently, and data were given as the mean ± S.E.M. The P-value was set at # P < 0.05, ## P < 0.01 versus the control group; *P < 0.05, **P < 0.01 versus the LPS group; ns, not significant versus the LPS group.

NF-κB and MAPKs are the two main TLR4 signaling axes. Immunoblotting was employed to measure the effect of CBDV on TLR4 signaling. As shown in **Figure 3**, CBDV inhibited LPS-induced phosphorylation of IKKβ and p65 as well as LPS-induced phosphorylation of JNK, ERK and p38 in a concentration-dependent manner. These results indicate that CBDV inhibits TLR4 signaling NF-κB and MAPK signaling axes.

**Figure 4:**
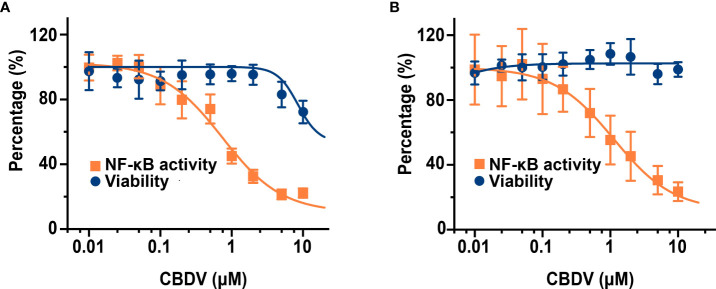
CBDV inhibits LPS-induced NF-κB activation in HEK Blue hTLR4 cells **(A)** and immunocompetent microglial BV-2 cells **(B)**. **(A)** HEK Blue hTLR4 cells, which overexpress human CD14, TLR4, and MD-2, were stimulated with LPS and indicated concentrations of CBDV. The NF-κB activity was determined by SEAP assay and the cellular viability was measured by CCK-8 Kit. **(B)** BV-2 NF-κB luciferase reporter cells were treated with LPS and indicated concentrations of CBDV. The NF-κB activity was determined by the Steady-Glo luciferase assay and the cellular viability was measured by crystal violet staining. All experiments were performed three times independently, and data were given as the mean ± S.E.M.

To further quantitatively investigate the effect of CBDV on TLR4 signaling NF-κB activity, SEAP assay based on the HEK Blue hTLR4 cells was performed. As shown in **Figure 4A**, CBDV inhibited LPS-induced NF-κB activation in a dose-dependent manner, with an IC_50_ of 1.4 ± 0.2 μM, while the cellular toxicity of CBDV on HEK Blue hTLR4 cells (IC_50_ = 23.3 ± 2.5 μM) was low. In addition to HEK-based NF-κB reporter cells, the effect of CBDV on NF-κB activity in BV-2 microglial cells was also examined. CBDV inhibited LPS-induced NF-κB activation in BV-2 cells in a dose-dependent manner with an IC_50_ of 1.7 ± 0.2 μM without apparent cellular toxicity within 10 μM (**Figure 4B**). These data clearly show that CBDV inhibits TLR4 signaling NF-κB activation.

**Figure 5:**
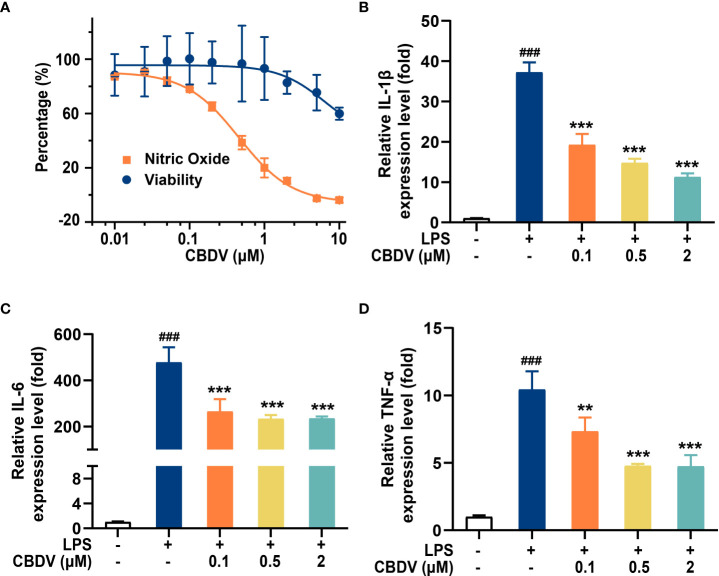
CBDV inhibits LPS-induced NO overproduction **(A)** as well as the pro-inflammatory factors IL-1β **(B)**, IL-6 **(C)**, and TNF-α **(D)** mRNAs expression. All the data represented the mean ± S.E.M.; the number of independent cell culture preparations = 3. The P-value was set at ### P < 0.001 versus the control group; **P < 0.01, ***P < 0.001 versus the LPS group.

The activation of TLR4 signaling promotes the over-production of pro-inflammatory factors, including NO, IL-1β, IL-6, and TNF-α. To validate the effect of CBDV on downstream inflammatory factors, NO assay and qPCR were performed. CBDV inhibited LPS-induced NO elevation with an IC_50_ of 0.5 ± 0.3 μM (**Figure 5A**). Moreover, CBDV suppressed LPS-induced IL-1β (**Figure 5B**), IL-6 (**Figure 5C**), and TNF-α (**Figure 5D**) mRNA expression in a dose-dependent manner. Together, these cellular data show that CBDV restrains LPS-induced activation of TLR4 signaling, therefore inhibiting the over-production of TLR4 downstream pro-inflammatory factors.

#### CBDV improves morphine-mediated analgesia

**Figure 6:**
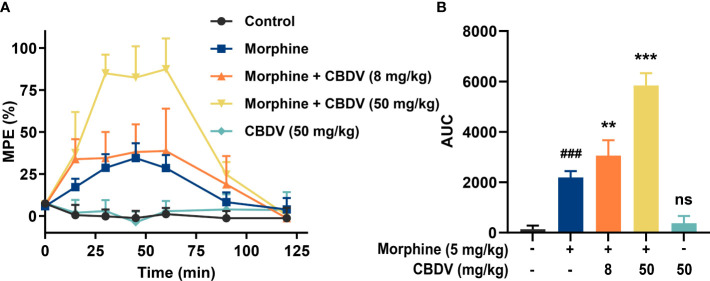
CBDV potentiates morphine-induced antinociception. **(A)** Intraperitoneal co-administration of morphine (5 mg/kg) and CBDV produced a significant potentiation of morphine hot plate analgesia. **(B)** The area under the curve (AUC) curves shown in panel **(A)**. Data were presented as the mean ± S.E.M. n = 5/group; The P-value was set at ### P < 0.001 versus the control group; **P < 0.01, ***P < 0.001 versus the morphine group; ns, not significant versus the control group.

Morphine induces glia activation and neuroinflammation *via* TLR4 (7, 14), and TLR4 antagonists have been found to enhance the morphine analgesic effect and attenuate morphine tolerance (42, 43). Hot plate assay was performed to test whether CBDV could potentiate morphine analgesia (**Figure 6A**). Morphine produced significant analgesia to the hot plate, compared to baseline. TLR4 antagonist CBDV was found to increase and prolong morphine analgesia in a dose-dependent manner (**Figure 6B**). However, CBDV had no effect on heat pain responsivity in the absence of co-administered morphine.

**Figure 7:**
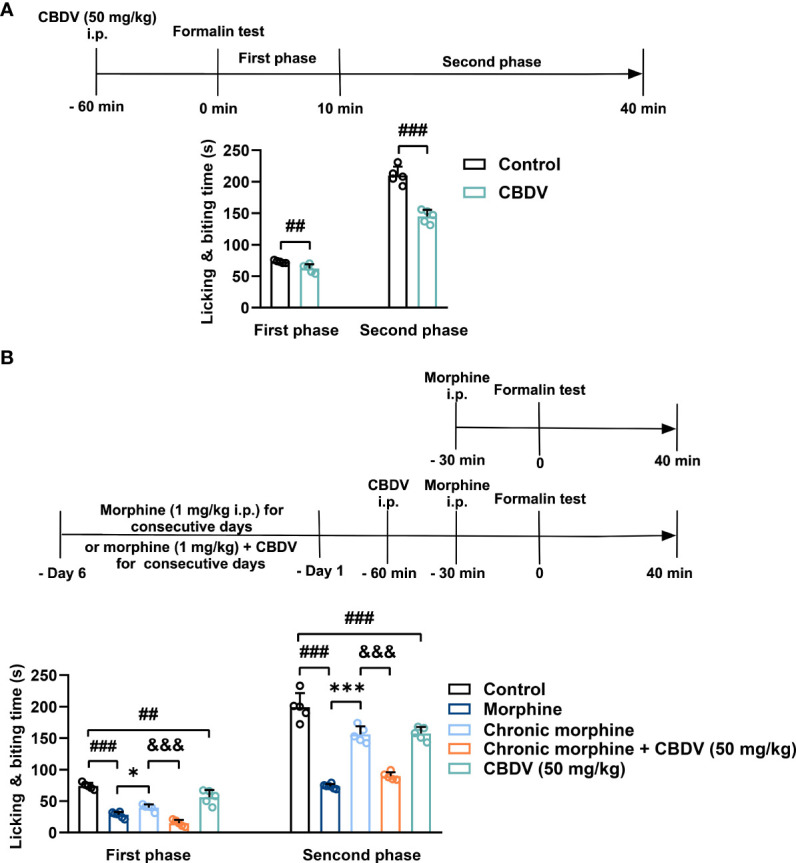
CBDV shows analgesic effect and attenuates morphine analgesic tolerance as measured by formalin test. **(A)** The analgesic effect of single administration of CBDV as assessed by formalin test. **(B)** The effect of CBDV on the analgesic activity of chronic morphine as measured by the formalin test. Data were presented as the mean ± S.E.M. n = 5/group; The P-value was set at ## P < 0.01, ### P < 0.001 versus the control group; *P < 0.05, ***P < 0.001 versus the morphine group; &&& P < 0.001 versus the chronic morphine group.

**Figure 8:**
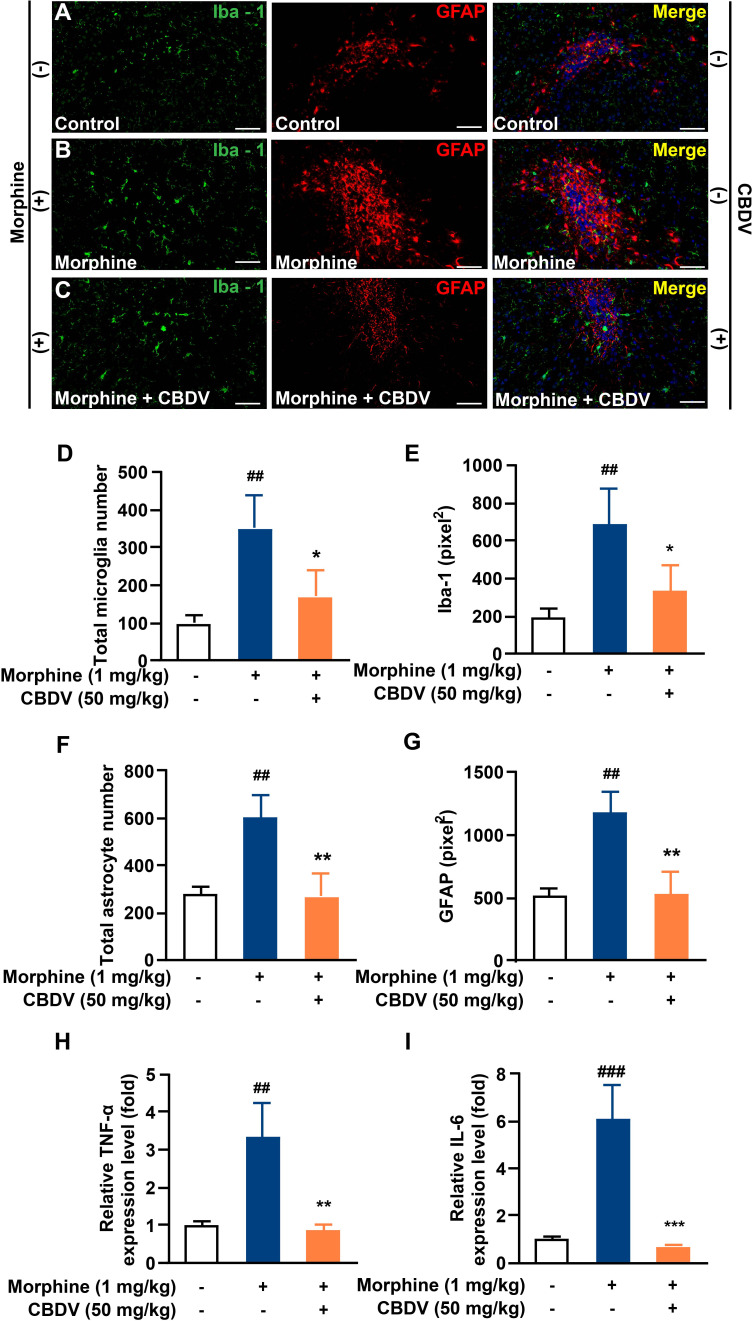
CBDV inhibits chronic morphine treatment-induced glial activation and pro-inflammatory factors IL-6 and TNF-α mRNA expression in NAc. **(A–C)** Representative double immunofluorescent staining images of Iba1 and GFAP for the control group **(A)**, morphine group **(B)**, and morphine + CBDV group **(C)**. NAc regions were collected following the final behavioral testing shown in **Figure 7B**. **(D, F)** The quantification of microglia **(D)** and astrocytes **(F)**. **(E, G)** The size of the microglia **(E)** and astrocytes **(G)**. **(H, I)** Total RNAs were extracted and qRT-PCR was performed to measure the expression of TNF-α **(H)** and IL-6 **(I)** in NAc. Scale bar: 200 μm. All the data represented mean ± S.E.M. ## P < 0.01, ### P < 0.001 versus the control group; *P < 0.05, **P < 0.01, ***P < 0.001 versus the morphine group.

In addition to hot plate assay, formalin test was also performed to evaluate the analgesic activity of CBDV and to assess the combinational effect of CBDV and morphine. The formalin test is a popular model of clinical pain, which includes both acute pain (0-10 min) and tonic pain (10-40 min). The first phase is attributed to C-fiber activation due to the peripheral stimulus by formalin, while the second phase appears to be associated with the inflammation response. CBDV showed analgesic effects in both the acute phase and tonic phase (**Figure 7A**). Compared to single administration, chronic morphine treatment induced analgesia tolerance as reflected in both phases of the formalin test. Moreover, co-administration of CBDV was found to attenuate tolerance to morphine analgesia (**Figure 7B**). To further analyze whether the attenuation of morphine tolerance by CBDV was associated with its inhibition of glial activation, nucleus accumbens (NAc) that is a key neural substrate for opioid-mediated pain modulation (44, 45), was dissected following the behavioral testing. The tissues were stained for microglia and astrocyte markers Iba-1 and GFAP, respectively (**Figures 8A–C**). Compared to the control, the number and size of microglia, as well as astrocyte in the NAc were elevated in the chronic morphine treated group. CBDV inhibited the activation of microglia (**Figures 8D, E**) and astrocytes (**Figures 8F, G**). Meanwhile, the tissue RNA extraction and qPCR were performed to examine the expression of pro-inflammatory factors. Chronic morphine treatment was found to increase pro-inflammatory factors TNF-α (**Figure 8H**) and IL-6 (**Figure 8I**) mRNAs expression in the NAc, which clearly shows that morphine induces a neuroinflammatory milieu. CBDV was found to inhibit morphine-induced TNF-α (**Figure 8H**) and IL-6 (**Figure 8I**) mRNAs expression in the NAc. In contrast, chronic morphine failed to activate glia in mPFC (**Supplementary Figure 1**) and VTA (**Supplementary Figure 2**) regions and CBDV did not affect TNF-α and IL-6 mRNAs expression in these regions. These results imply that CBDV improves morphine-mediated analgesia by specifically inhibiting morphine-induced glial activation and pro-inflammatory factors expression in NAc.

### Discussion

Since being isolated in 1969 (46), the underlying mechanisms of CBDV actions are little known. It should be noted that cannabinoid receptors are not the primary targets of CBDV owing to the low binding affinity (47, 48). Considering that CBDV has good BBB permeability (20, 21), it would be interesting to investigate how CBDV regulates central immunity, which is mainly mediated by CNS resident innate immune cells microglia and astrocytes. TLR4 is abundantly expressed in glia and is the key PRR of the innate immune system, which detects PAMPs, DAMPs and XMAPs (5). Therefore, the interaction of CBDV with TLR4 co-receptor MD2, which is responsible for the recognition of TLR4 ligand, was investigated. Herein, *in vitro* quenching titrations of MD2 intrinsic fluorescence showed the direct binding of CBDV to MD2. CETSA confirmed that MD2 was the endogenous target of CBDV. The RMSF analysis indicated that CBDV destabilized MD2, which is consistent with CETSA data. Cellular characterizations found that CBDV inhibited TLR4 signaling NF-κB and MAPKs axes, therefore suppressing neuroinflammation. This study identified MD2 as a direct target of CBDV, which at least in part accounts for its anti-neuroinflammatory activity. It should be acknowledged that there may be other unknown targets, which are worth further investigation.

Our previous work demonstrated that morphine bound to MD-2, induced TLR4 oligomerization, and activated TLR4 signaling (7). Morphine induces glial activation and neuroinflammation, which compromises morphine analgesia and contributes to morphine tolerance (42, 43). Therefore, TLR4 would be a novel target for therapeutic development to improve the current opioid-based pain management therapies. In this study, TLR4 antagonist CBDV was found to increase and prolong morphine analgesia in a dose-dependent manner. Moreover, CBDV attenuated morphine tolerance. Furthermore, the *in vivo* results show that CBDV improved morphine-mediated analgesia by specifically inhibiting chronic morphine-induced glial activation and pro-inflammatory factors expression in the NAc. It should be noted that CBDV alone showed no analgesic activity as tested by the hot plate assay while CBDV showed analgesic effects in both the acute phase and tonic phase as measured by the formalin test. This is not surprising considering that these two behavioral models of nociception show contrast different cellular and molecular mechanisms of pain (49, 50). Further elucidation is needed for a better understanding of these different behavioral responses of CBDV.

Considering the facts that morphine is the most commonly used opioid analgesic in the clinic (51) and the opioid crisis has been a significant public health burden (52), there is an urgent need to boost the analgesic activity, reduce the used dose and prevent the tolerance side effect of morphine. Previous proof-of-concept studies have demonstrated that TLR4 small-molecule antagonists would provide a nonconventional avenue to improve the clinical efficacy of opioids and possibly improve safety (7, 53). Consequently, numerous TLR4 antagonists have been developed (54, 55). However, few of them could cross BBB (16). This study adds CBDV, which has good BBB penetrability, as a potent TLR4 antagonist. Although CBDV is safe (18), the solubility of CBDV is poor, which may limit the therapeutic applications of CBDV by its low bioavailability (56). Incorporating CBDV into a novel drug delivery system is hopefully to boost its bioavailability, prolong its half-life, and enhance the therapeutic efficacy.

**Figure 9:**
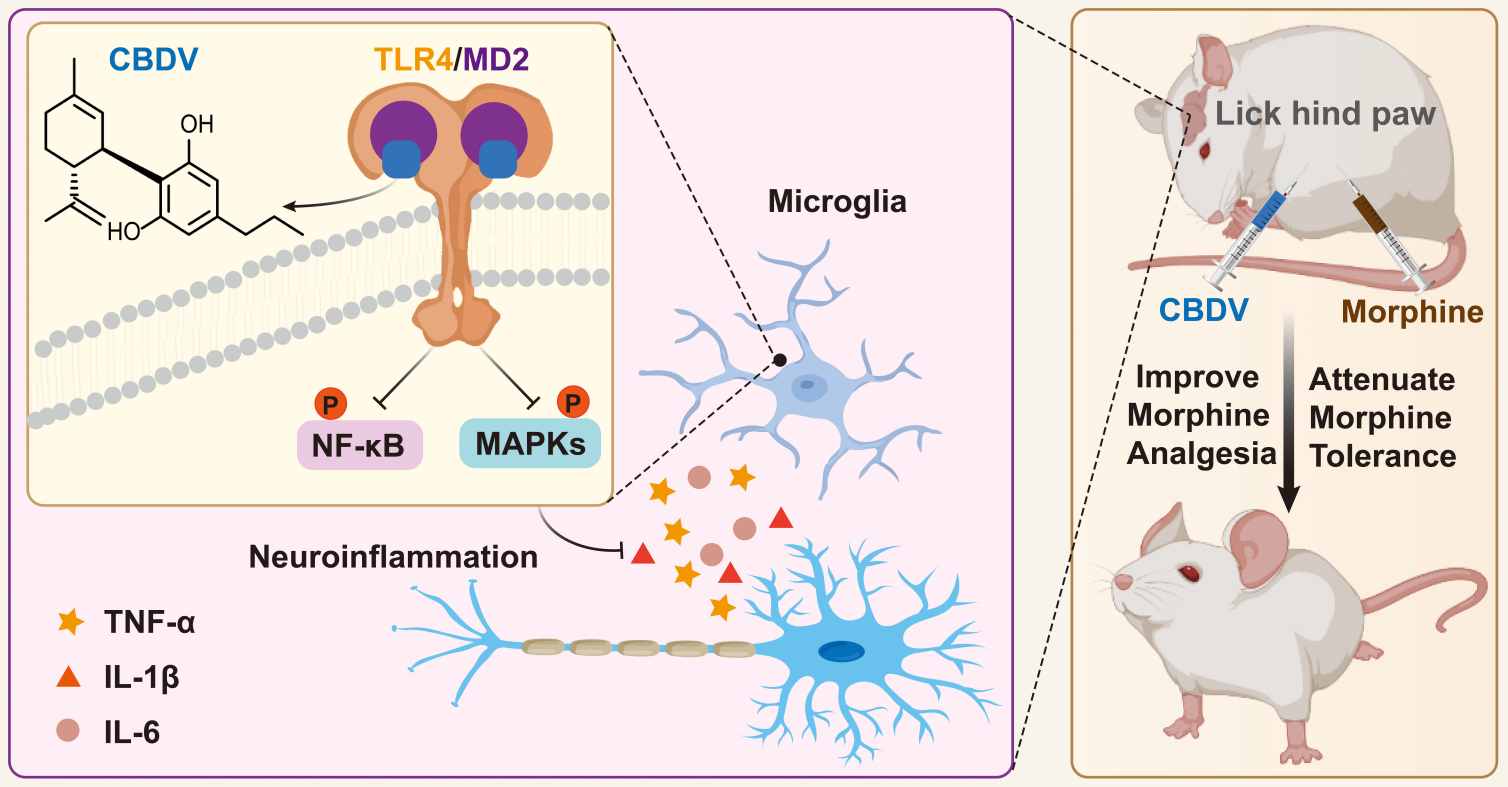
A schematic illustration of the role of CBDV in modulating TLR4 signaling *via* targeting MD2. CBDV binds to TLR4 co-receptor MD2, inhibits TLR4 signaling NF-κB and MAPKs, and suppresses downstream pro-inflammatory cytokines TNF-α, IL-1β and IL-6. By inhibiting microglial-induced neuroinflammation, CBDV improves morphine-mediated analgesia.

In summary, this study clearly confirms that MD2 is a direct binding target of CBDV for the anti-neuroinflammatory effect and implies that CBDV is a TLR4 antagonist, which can partially explain its interference of innate immune function in CNS. Furthermore, CBDV improves morphine-mediated analgesia by inhibiting morphine-induced glial activation and pro-inflammatory factors expression (**Figure 9**). The results imply that CBDV could be a potential therapeutic agent for improving morphine-mediated analgesia.

## Data availability statement

The original contributions presented in the study are included in the article/**Supplementary Material**. Further inquiries can be directed to the corresponding authors.

## Ethics statement

The animal study was reviewed and approved by The Institutional Animal Care and Use Committee (IACUC) of Changchun Institute of Applied Chemistry, Chinese Academy of Sciences (2022-0090).

## Author contributions

XiW designed the experiments. XuW, CL, YW, TZ and SW performed the experiments, acquired and analyzed data. XuW, CL and XiW wrote the manuscript. YJ and XiW edited the manuscript. All authors read and approved the final manuscript.

## Funding

This work was supported by Brain Science and Brain-Like Intelligence Technology Program (2021ZD0203003), Beijing National Laboratory for Molecular Sciences (BNLMS202108), and the Chinese Academy of Sciences (CAS) Pioneer Hundred Talents Program.

## Acknowledgments

Computing time was supported by National Supercomputer Center in Tianjin.

## Conflict of interest

The authors declare that the research was conducted in the absence of any commercial or financial relationships that could be construed as a potential conflict of interest.

## Publisher’s note

All claims expressed in this article are solely those of the authors and do not necessarily represent those of their affiliated organizations, or those of the publisher, the editors and the reviewers. Any product that may be evaluated in this article, or claim that may be made by its manufacturer, is not guaranteed or endorsed by the publisher.
