# Supplementary material for: Cannabidivarin alleviates neuroinflammation by targeting TLR4 co-receptor MD2 and improves morphine-mediated analgesia
Source: Front Immunol. 2022 Aug 10;13:929222. doi: 10.3389/fimmu.2022.929222 (PMC9399816; doi:10.3389/fimmu.2022.929222)
Supplement: Supplementary file 1 [file DataSheet_1.docx]

Supplementary Material

# Supplementary Figures and Tables

## Supplementary Figures


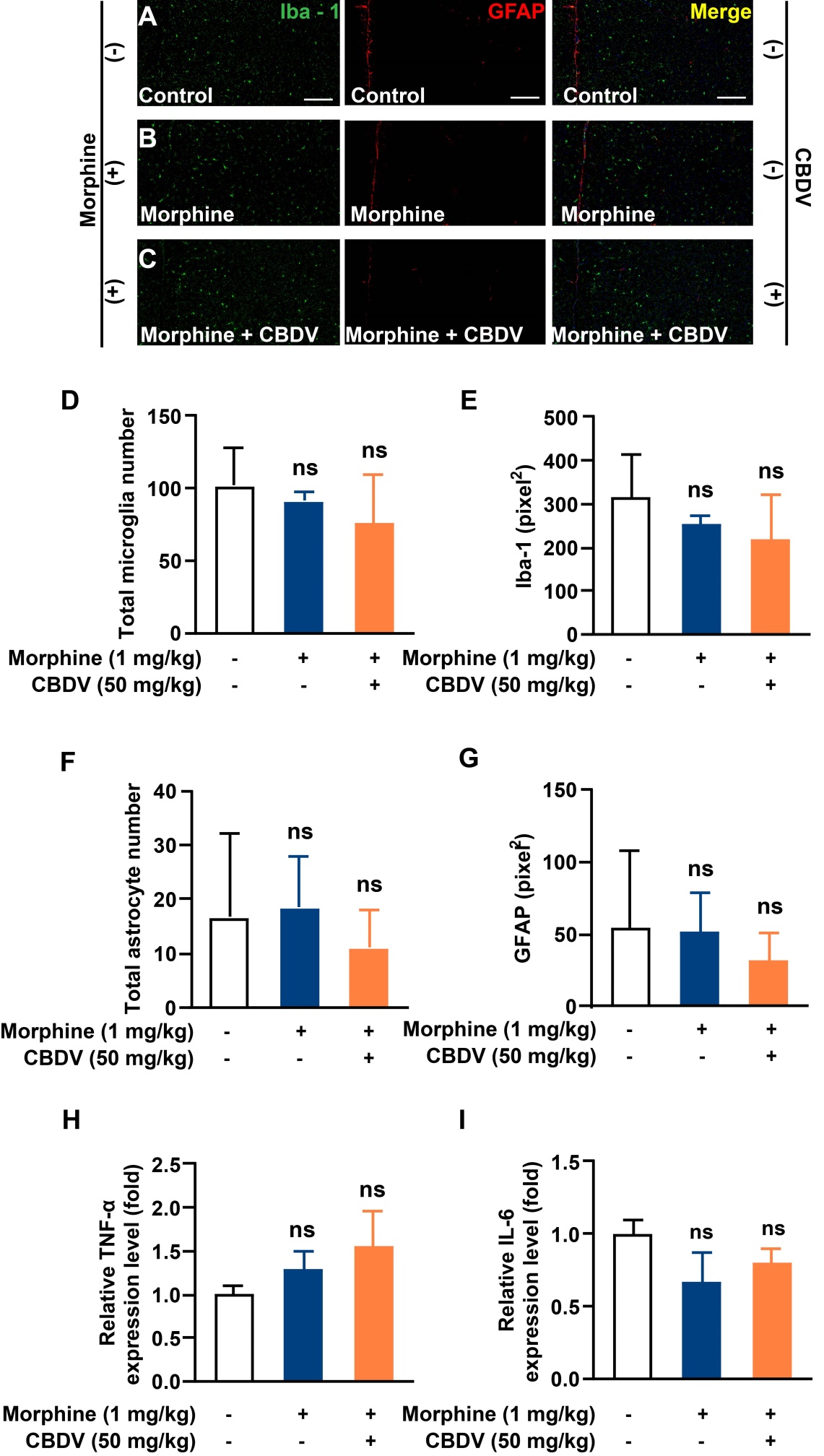


**Supplementary Figure 1**. **CBDV does not inhibit chronic morphine treatment-induced glial activation and pro-inflammatory factors IL-6 and TNF-α mRNA expression in mPFC region.** (A-C) Representative double immunofluorescent staining images of Iba1 and GFAP for the control group (A), morphine group (B) and morphine + CBDV group (C). mPFC regions were dissected following the final behavioral testing shown in Figure 7B. (D, F) The quantification of microglia (D) and astrocytes (F). (E, G) The size of the microglia (E) and astrocytes (G). (H, I) Total RNAs were extracted and qRT-PCR was performed to measure the expression of TNF-α (H) and IL-6 (I) in mPFC. Scale bar: 200 μm. All the data represented mean ± S.E.M. ns, not significant.


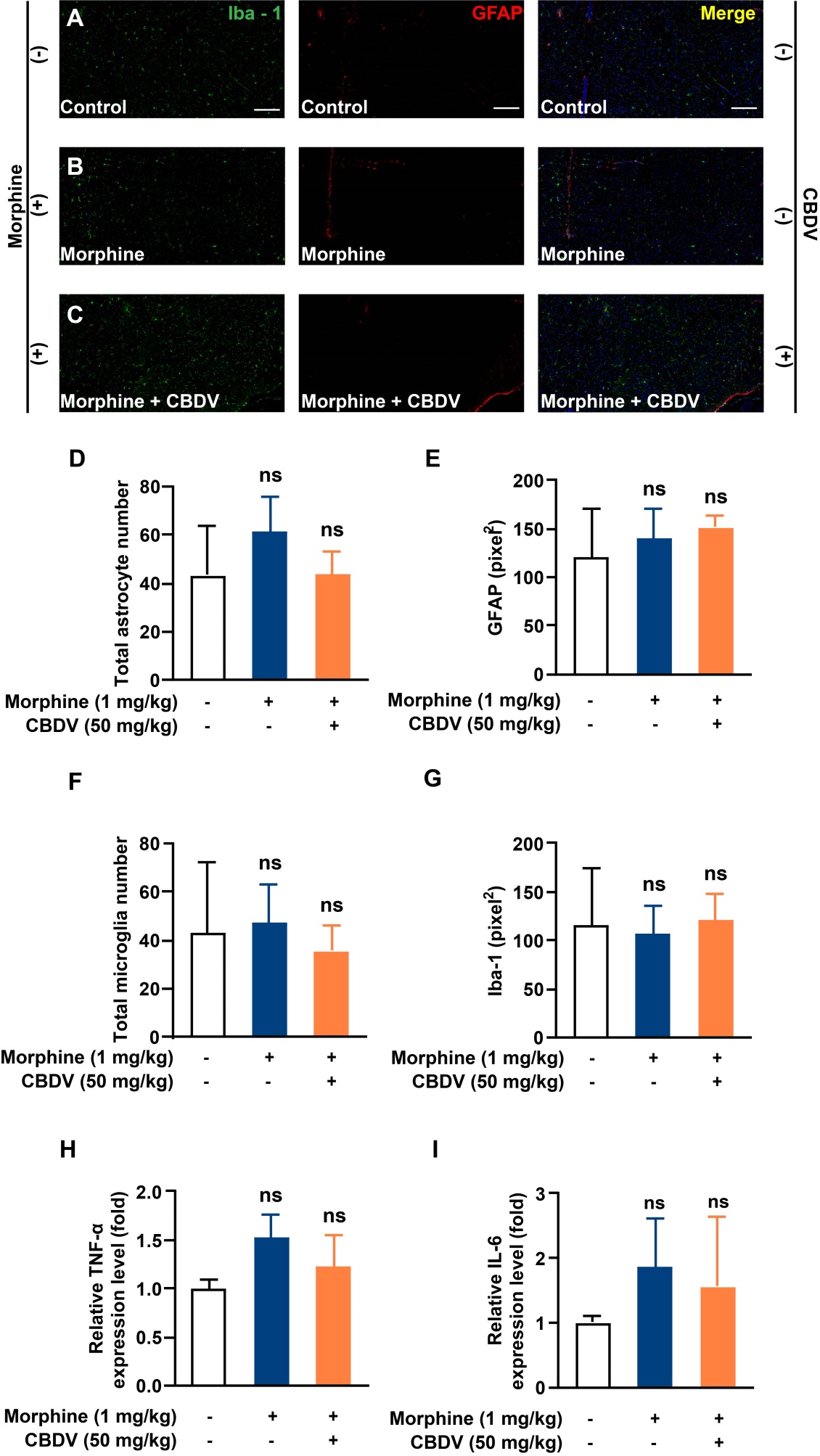


**Supplementary Figure 2**. **CBDV does not inhibit chronic morphine treatment-induced glial activation and pro-inflammatory factors IL-6 and TNF-α mRNA expression in VTA region.** (A-C) Representative double immunofluorescent staining images of Iba1 and GFAP for the control group (A), morphine group (B), and morphine + CBDV group (C). VTA regions were collected following the final behavioral testing shown in Figure 7B. (D, F) The quantification of microglia (D) and astrocytes (F). (E, G) The size of the microglia (E) and astrocytes (G). (H, I) Total RNAs were extracted and qRT-PCR was performed to measure the expression of TNF-α (H) and IL-6 (I) in VTA. Scale bar: 200 μm. All the data represented mean ± S.E.M. ns, not significant.
